# Supplementary material for: Safety and efficacy studies of CRISPR-Cas9 treatment of sickle cell disease highlights disease-specific responses
Source: Mol Ther. 2024 Jul 22;32(12):4337–52. doi: 10.1016/j.ymthe.2024.07.015 (PMC11638826; doi:10.1016/j.ymthe.2024.07.015)
Supplement: Document S1. Figures S1–S6 and Tables S8–S12 [file mmc1.pdf]

YMTHE, Volume *32*

Supplemental Information

Safety and efficacy studies of CRISPR-Cas9 treatment of sickle cell disease highlights disease-specific responses

Giacomo Frati, Megane Brusson, Gilles Sartre, Bochra Mlayah, Tristan Felix, Anne Chalumeau, Panagiotis Antoniou, Giulia Hardouin, Jean-Paul Concordet, Oriana Romano, Giandomenico Turchiano, and Annarita Miccio

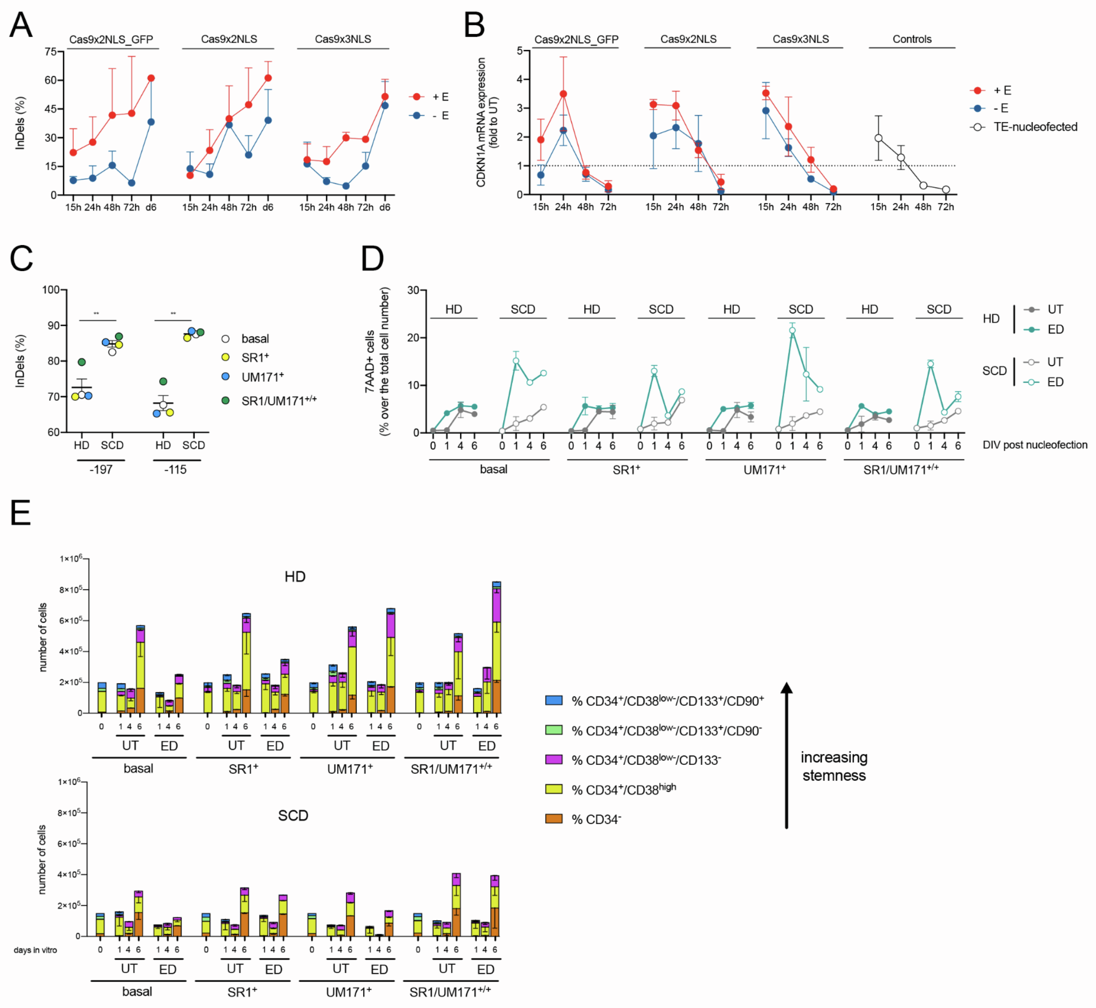

Figure S1. Optimization of the genome editing procedure in primary HSPCs. (**A**) Time course analysis of InDel frequency measured in CB-derived HD HSPCs treated with three different Cas9 RNPs (Cas9x2NLS_GFP, Cas9x2NLS, Cas9x3NLS) in the presence (+E, red line) or in the absence (-E, blue line) of the electroporation enhancer. We used the -197 sgRNA. (**B**) Time course analysis of *CDKN1A* mRNA expression in CB-derived HD HSPCs treated with three different Cas9 RNP nucleases (Cas9x2NLS_GFP, Cas9x2NLS, Cas9x3NLS) in the presence (+E, red line) or in the absence (-E, blue line) of the electroporation enhancer. Data are reported as mean ± SEM of n=2 run in triplicate. **p<0.01; paired t-test. (**C**) GE efficiency in cord blood-derived (HD) and non-mobilized (SCD) CD34+ cells measured by Sanger sequencing followed by TIDE analysis in samples edited with two different *HBG*-targeting sgRNA (the -197 sgRNA or the -115 sgRNA targeting the -115 region of the *HBG* promoters[2]). Each colored dot indicates a different culture condition. Data are reported as mean ± SEM of 4 replicates. (**D**) Time course analysis of the percentage of dead cells (measured as 7AAD^{+} cells) in edited (ED, green line) and control samples (UT, grey line) in HD- (filled dots) vs SCD (empty dots) patient-derived HSPCs cultured in the presence (+) or in the absence (-) of SR1 and/or UM171. Data are reported as mean ± SEM of 2 replicates. (**E**) Bar plots showing the cell composition of HD- (top panel) and SCD (bottom panel) patient-derived HSPCs cultured in the presence (+) or in the absence (-) of SR1 and/or UM171. We defined 5 populations with increasing stemness properties (CD34- cells, CD34+/CD38high cells, CD34+/CD38low-/CD133- cells, CD34+/CD38low-/CD133+/CD90- cells, and CD34+/CD38low-/CD133+/CD90+ cells). Starting from day 4, compared to HD samples, SCD cells had a larger fraction of more differentiated CD34^{-} cells and the CD34^{+}/CD38low^{-}/CD133^{+}/CD90^{+} cell

population (more enriched in HSCs) disappeared in SCD samples. In fact, this latter fraction is still present in HD cells at day 4, although it is smaller compared to the beginning of the culture. However, it is worth to notice that since the beginning SCD samples have a higher proportion of $CD34^-$ cells and a lower percentage of $CD34^+$/CD38low$^-$/$CD133^+$/$CD90^+$ cells, which could be ascribed to the cell origin (patient adult vs HD cord blood HSPCs). Data are reported as mean ± SEM of 2 replicates.

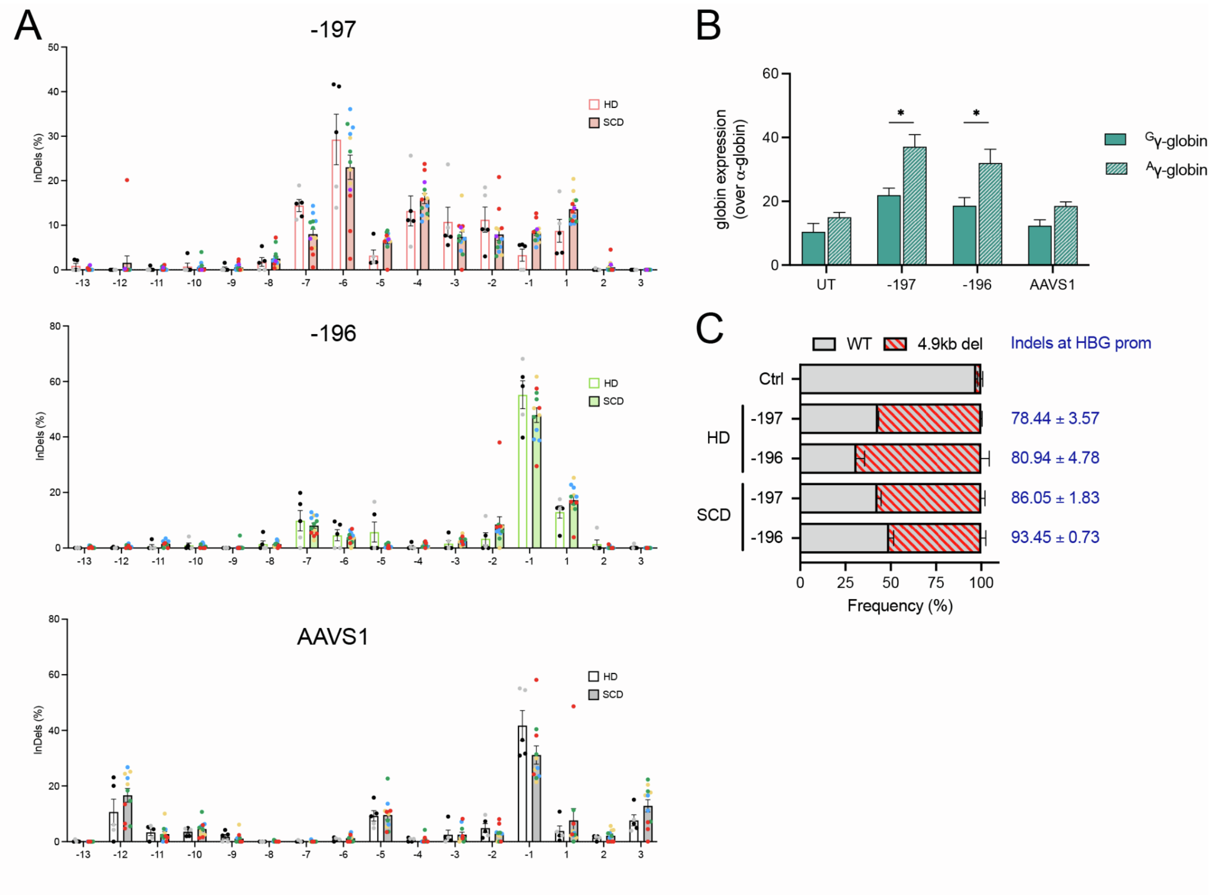

Figure S2. Editing profiles in primary SCD and HD HSPCs. (**A**) Single frequency of individual InDels in healthy donor (HD) and sickle cell disease (SCD) HSPCs treated with the -197, -196 and AAVS1 sgRNAs. InDel frequency was evaluated by Sanger sequencing followed by TIDE analysis. n= 2 donors for HD; n=5 donors for SCD. (**B**) Expression of $^{G}\gamma$- and ^{A} γ-globin chains measured by RP-HPLC in pooled SCD BFU-E colonies derived from untreated (UT) HSPCs and HSPCs treated with the -197, -196 and AAVS1 sgRNA. Data are expressed as mean ± SEM n=4, *p<0.05 Mann-Whitney test. (**C**) Frequency of 4.9-kb deletion as measured by ddPCR in healthy donor (HD) and sickle cell disease (SCD) HSPCs. We also reported mean ± SEM values of the overall InDels measured by Sanger sequencing and TIDE analysis at the *HBG1/2* promoters. UT and AAVS1-treated cells served as negative control (Ctrl).

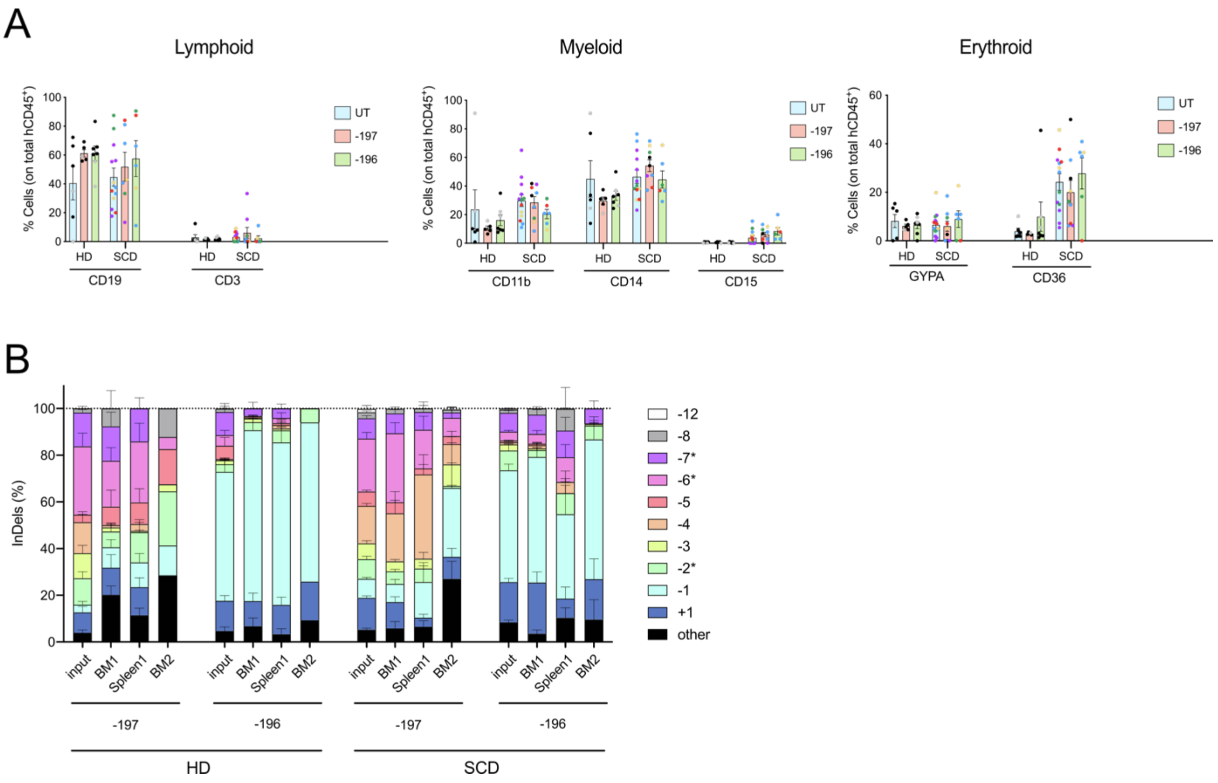

Figure S3. Multilineage differentiation and editing profiles in primary SCD and HD HSPCs. (**A**) Frequency of human T (CD3) and B (CD19) lymphoid, myeloid (CD14, CD15 and CD11b) and erythroid (CD36, GYPA) cells in the spleen of mice transplanted with control and edited HSPCs. (**B**) Frequency (expressed as %) of individual InDel events in the input populations and in BM- and spleen-derived human CD45+ cells edited with the −197 and −196 sgRNAs, as evaluated by Sanger sequencing and TIDE analysis. The numbers 1 and 2 after the organs' name indicate primary and secondary recipients, respectively. * indicate putative microhomology associated events.

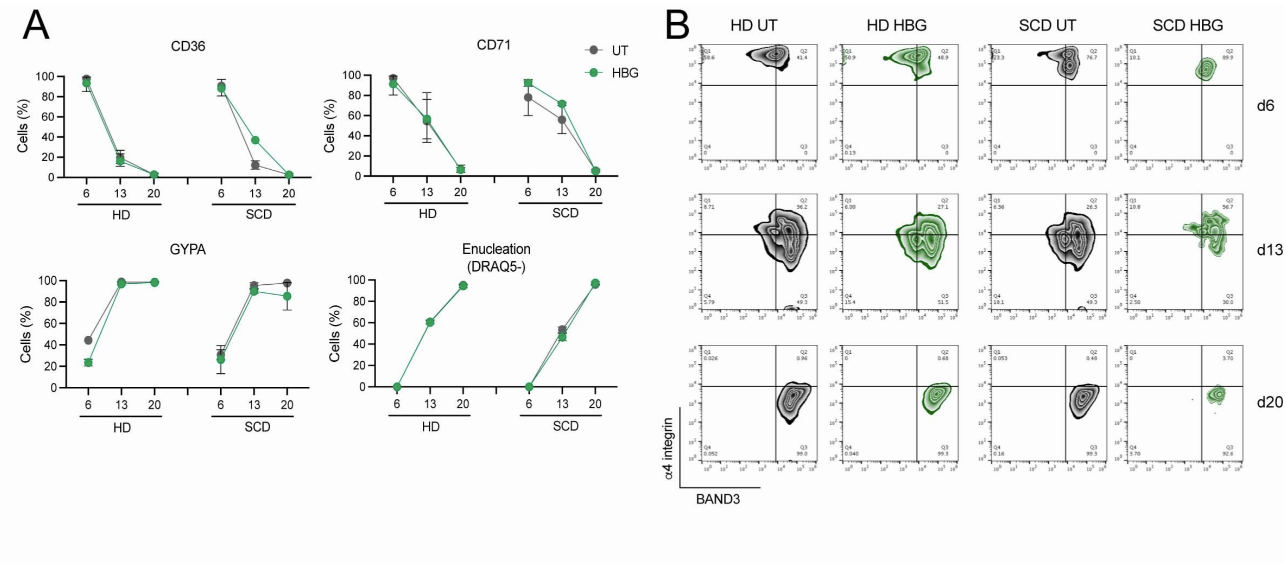

Figure S4. Erythroid differentiation of edited HSPCs. (**A**) Flow cytometry analysis of early (CD36 and CD71) and late (Glycophorin A GYPA) erythroid markers, and enucleation rate (measured as frequency of DRAQ5⁻ cells) in *ex vivo* differentiated erythrocytes. Data are plotted as means ± SEM (n=2-6). (**B**) Representative flow-cytometry plots showing the expression of BAND3 and α4 integrin in 7AAD⁻ GYPA⁺ cells at day (d) 6, 13 and 20 of *ex vivo* erythroid differentiation of the BM-repopulating hCD45⁺ cells. Edited samples (HBG) are highlighted in green and untreated (UT) samples in grey.

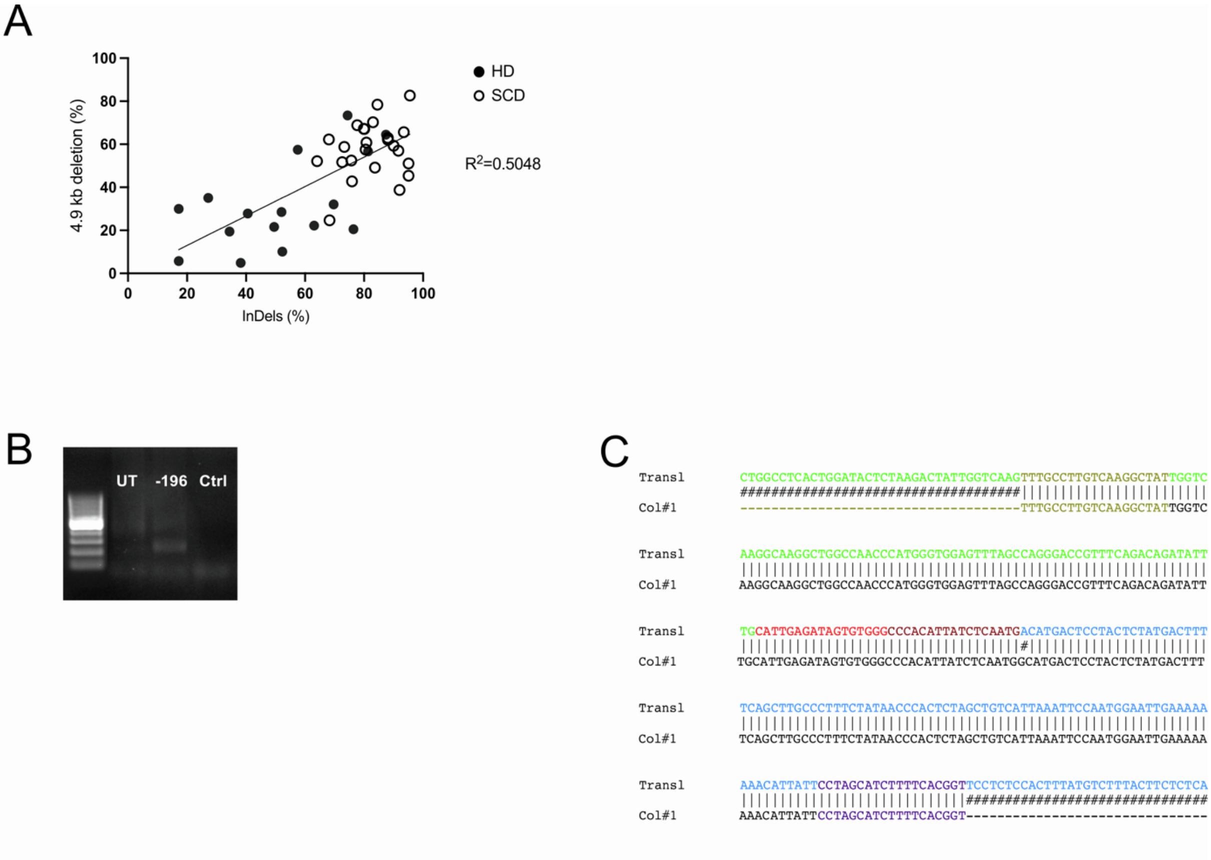

Figure S5. Chromosomal rearrangements in edited primary hematopoietic cells. (**A**) Correlation between InDel and 4.9-kb deletion frequency in SCD and HD primary hematopoietic cells *in vitro* and *in vivo*. (**B**) Agarose gel image showing a band corresponding to the PCR product obtained using primers surrounding the putative translocation junctions in untreated (UT) SCD HSPCs or SCD HSPCs treated with the sgRNA -196 (-196). As control, we performed the PCR reaction in the absence of DNA (ctrl). (**C**) Alignment between the *in silico* predicted translocation (Trans1) and the sequence of the PCR product showed in A (Col#1). Green and blue identify the *HBG* promoter and off-target on chromosome 12 sequence, respectively. We indicated in red and brown the sequences of the -196 sgRNA on-target site and of the off-target site on chromosome 12 (without the 3 last nucleotides downstream of the cleavage site).

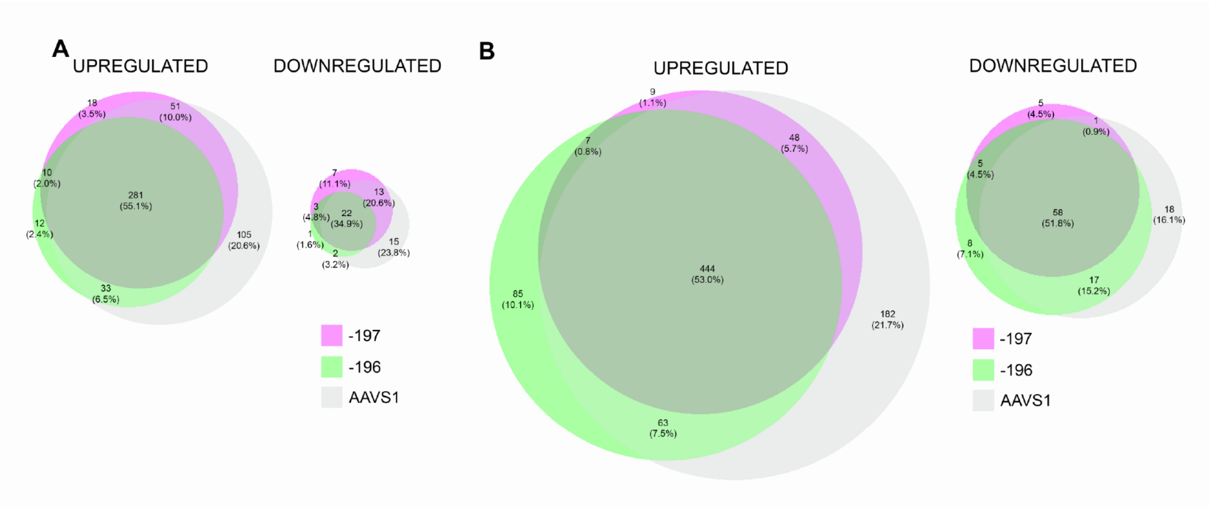

Figure S6. DEGs in primary SCD and HD HSPCs treated with control and *HBG*-targeting sgRNAs. (**A**) Venn diagrams showing the differentially expressed genes (DEGs) upregulated and downregulated in HD samples upon treatment with the sgRNA -197 (pink), -196 (green) and AAVS1 (grey). (**B**) Venn diagrams showing the DEGs upregulated and downregulated in SCD samples upon treatment with the sgRNA -197 (pink), -196 (green) and AAVS1 (grey). In all diagrams, circle size correlates with the number of DEGs. Graphs were generated using BioVenn[63].

Table S1. List of on-target, off-targets and natural break sites (-196 gRNA).

Table S2. List of on-target, off-targets and natural break sites (-197 gRNA).

Table S3. Differentially expressed genes in SCD vs HD HSPCs

Table S4. Differentially expressed genes in HD HSPCs treated with sgRNA -197 vs HD UT HSPCs, Differentially expressed genes in HD HSPCs treated with sgRNA -196 vs HD UT HSPCs and Differentially expressed genes in HD HSPCs treated with sgRNAAAVS1 vs HD UT HSPCs

Table S5. Differentially expressed genes in SCD HSPCs treated with sgRNA -197 vs SCD UT HSPCs, Differentially expressed genes in SCD HSPCs treated with sgRNA -196 vs SCD UT HSPCs and Differentially expressed genes in SCD HSPCs treated with sgRNA AAVS1 vs SCD UT HSPCs

Table S6. Hallmark gene set enrichment of up-regulated genes in SCD treated vs SCD UT (pathways shown in Figure 4D are in bold) and hallmark gene set enrichment of up-regulated genes in HD treated vs HD UT (pathways shown in Figure 4D are in bold)

Table S7. List of SCD-specific, HD-specific and common DEGs and hallmark gene set enrichment of genes specifically up-regulated in SCD and HD samples.

Table S8. Primers used to detect InDels events. F, forward primer; R, reverse primer.

**Amplified region**		Sequence (5' to 3')
***HBG1* + *HBG2* promoters**	F	AAAAACGGCTGACAAAAGAAGTCCTGGTAT
	R	ATAACCTCAGACGTTCCAGAAGCGAGTGTG
***AAVS1* site**	F	CAGCACCAGGATCAGTGAAA
	R	CTATGTCCACTTCAGGACAGCA
**Off-target Chr9**	F	GACAGCAGAGAGTTGGATTTAGCC
	R	CCCATTCTCCCTCTCTTCTTCTTG
**Off-target Chr12**	F	TCAGTGGGAGGAGTATTTGAAGGC
	R	CTAGTAGGGTCCCTTCCTCTACTT

Table S9. Primers used for ddPCR. F, forward primer; R, reverse primer; P, probe

**Amplified region**	**F/R**	**Sequence (5' to 3')**
***HBG1-HBG2* intervening region**	**F**	ACGGATAAGTAGATATTGAGGTAAGC
	**R**	GTCTCTTTCAGTTAGCAGTGG
	**P**	FAM-ACTGCGCTGAAACTGTGGTCTTTATGA
hALB	**F**	ACTCATGGGAGCTGCTGGTT
	**R**	GCTGTCATCTCTTGTGGGCTG
	**P**	VIC-CCTGTCATGCCCACACAAATCTCTCC
HBG1-Chr12 translocation junction (#1)	**F**	TTTGCCTTGTCAAGGCTAT
	**R**	ACCGTGAAAAGATGCTAGG
	**P**	FAM-AGGCAAGGCTGGCCAACC
hRAD1	**F**	CCTTCAGCTCTGTGGTGACG
	**R**	CCCTTCTCAGCAAAGTCCCTG
	**P**	HEX-AGCGAGGCGGCTCCGAGGAA

Table S10. Primers used to detect Cas9-mediated translocation by CAST-seq.

**Primer name**	**Sequence (5' to 3')**
**Bait For**	GCCTCACTGGATACTCTAAG
**Decoy ForR**	ATTAGCAGTATCCTCTTGGG
**Decoy ForF**	TATAGCCTTTGCCTTGTTCC
**Nested For**	GACTGGAGTTCAGACGTGTGCTCTTCCGATCTCCAACCCATGGGTGGAGTTTAG
**Bait Rev**	ACGGCTGACAAAAGAAGTC
**Decoy Rev**	TGCAAATATCTGTCTGAAACG
**Nested Rev**	GACTGGAGTTCAGACGTGTGCTCTTCCGATCTGAATCGGAACAAGGCAAAGGC

Table S11. Primers used for regular PCR for the identification of inter-chromosomal translocation.

Putative Translocation	Combination		Sequence (5' to 3')
HBG-Chr12 translocation junction	***#1***	*HBG*_Fw	TTTGCCTTGTCAAGGCTAT
		Chr12_Fw	ACCGTGAAAAGATGCTAGG
	#2	*HBG*_Fw	TTTGCCTTGTCAAGGCTAT
		Chr12_Rev	ATCTCCCTTCCACTCAGAT
	#3	*HBG*_Rev	GAAGGAAACTAGCTAAAGGGA
		Chr12_Fw	ACCGTGAAAAGATGCTAGG
	#4	*HBG*_Rev	GAAGGAAACTAGCTAAAGGGA
		Chr12_Rev	ATCTCCCTTCCACTCAGAT
HBG-Chr9 translocation junction	***#1***	*HBG*_Fw	TTTGCCTTGTCAAGGCTAT
		Chr9_Fw	CCCTATTGCCGGCTCAACAG
	#2	*HBG*_Fw	TTTGCCTTGTCAAGGCTAT
		Chr9_Rev	GGAGAGCTATGATGACAAGTGG
	#3	*HBG*_Rev	GAAGGAAACTAGCTAAAGGGA
		Chr9_Fw	CCCTATTGCCGGCTCAACAG
	#4	*HBG*_Rev	GAAGGAAACTAGCTAAAGGGA
		Chr9_Rev	GGAGAGCTATGATGACAAGTGG

Table S12. Primers used for RT-qPCR. F, forward primer; R, reverse primer.

Amplified region	F/R	Sequence (5' to 3')
CDKN1A	F	CAGCATGACAGATTTCTACCACTC
	R	CTCGCGCTTCCAGGACTG
GAPDH	F	GAAGGTGAAGGTCGGAGT
	R	GAAGATGGTGATGGGATTTC
HBG1/2	F	CCTGTCCTCTGCCTCTGCC
	R	GGATTGCCAAAACGGTCAC
HBB	F	AAGGGCACCTTTGCCACA
	R	GCCACCACTTTCTGATAGGCAG
HBD	F	CAAGGGCACTTTTTCTCAG
	R	AATTCCTTGCCAAAGTTGC
HBA	F	CGGTCAACTTCAAGCTCCTAA
	R	ACAGAAGCCAGGAACTTGTC
